# How work hours affect well-being: A target trial emulation

**DOI:** 10.1371/journal.pone.0350816

**Published:** 2026-06-08

**Authors:** Ballerina X. S. Chong, Chris G. Sibley, Joseph A. Bulbulia

**Affiliations:** 1 School of Psychology, Victoria University of Wellington, Wellington, New Zealand; 2 School of Psychology, University of Auckland, Auckland, New Zealand; 3 Department of Linguistic and Cultural Evolution, Max Planck Institute for Evolutionary Anthropology, Leipzig, Germany; Guangxi Normal University, CHINA

## Abstract

Studies link longer work hours to multiple dimensions of well-being, but correlations do not show what would happen if hours changed. Target-trial emulation addresses this problem by specifying the experiment we would like to run and then approximating it with observational data. Using three annual waves of the New Zealand Attitudes and Values Study (NZAVS, *N* = 24,579; 2020–2023), we estimate how 28 well-being outcomes would differ if the same cohort of pre-retirement adults worked 10 more or 10 fewer hours per week than observed. We compare what would happen if weekly hours shifted up by 10 or down by 10 with what actually occurred, after accounting for dropout, using machine-learning methods to adjust for baseline differences. Increasing work hours by 10 most clearly raises fatigue and reduces sleep; body mass index (BMI) and perceived physical health also shift adversely but are more sensitive to residual confounding, while perceived support increases slightly but remains confounding-sensitive. Decreasing work hours by 10 most clearly lowers fatigue; BMI and perceived physical health also shift favourably but are likewise more sensitive to residual confounding. Most outcomes show little movement under either policy, and the downward shift is better supported by the data. Naive baseline associations are broader, larger, and sometimes reversed in sign, whereas sensitivity analyses (*E*-values) indicate that the clearest fatigue effects are robust to moderately strong residual confounding. Under the stated assumptions, work-hour shifts affect recovery and perceived physical health more than broad well-being.

## Introduction

Investigators often treat longer work hours as a threat to well-being, but the evidence is uneven. Resource-depletion models claim that extended work erodes time, energy, and self-regulatory capacity [1–3]. Consistent with this view, studies link longer hours to greater fatigue and psychological distress [4–8]. Yet studies report mixed findings for life satisfaction, physical health, and health behaviours [9–16]. This ambiguity is not surprising: people who work longer hours differ from those who work fewer in ways that also shape well-being, including occupational class, income, health, and family structure. Correlations therefore do not show what would happen if work hours changed, and these shared causes can make long hours appear either more harmful or more beneficial than they are.

**Fig 1:**
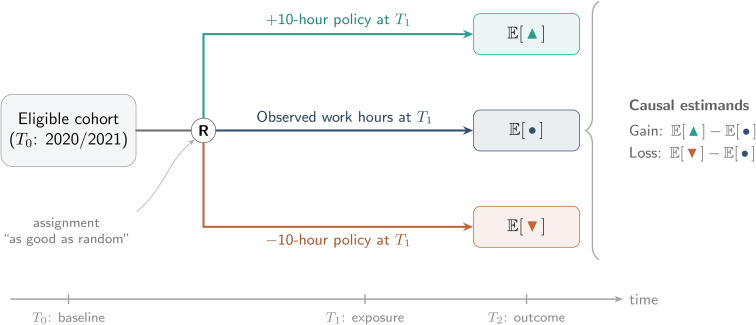
Conceptual target trial and its three policy-defined states. This figure shows the experiment we would like to run but cannot directly conduct: starting from a single baseline cohort (*T*_*0*_, 2020/2021), we compare later well-being under a + 10-hour policy at *T*_*1*_ (upward triangle), the identity policy that leaves work hours unchanged (filled circle), and a −10-hour policy at *T*_*1*_ (downward triangle). The causal estimands are the two population-average contrasts between each shifted policy and the identity policy under a no-censoring intervention. In the observational emulation, participants are not randomly assigned to these policies, so causal interpretation instead relies on exchangeability after baseline adjustment; we assess the remaining risk of residual confounding with sensitivity analyses.

A causal effect compares the same population under alternative, clearly defined strategies [17–20]. For work hours, we ask how the same baseline cohort would differ under alternative policies, such as working 10 more or 10 fewer hours per week than under their current regime. Fig 1 illustrates the conceptual target trial. After adjusting for baseline covariates (including baseline work hours and all baseline outcomes), we project the cohort into three conditions: a + 10-hour policy, the observed course corrected for loss to follow-up, and a −10-hour policy. The causal estimands are the two population-average contrasts between each shifted policy and the observed course.

An ideal experiment would randomly assign people from the same cohort to work-hour policies and compare their later well-being. If randomisation and control were to succeed, average responses under each policy would identify the corresponding population averages, and contrasts between these averages would estimate the corresponding causal effect for the population. Randomised controlled experiments are generally not feasible for interventions on work hours. Standard regressions using observational data, even longitudinal data, do not usually recover these effects. Even when confounding control is possible, regression coefficients alone do not define clear population-level intervention contrasts. Moreover, without a clearly defined treatment start, observational analyses are also vulnerable to selection biases [18,21,22]. Target-trial emulation addresses these problems by separating the causal question from the statistical estimation and defining treatment initiation [23,24]. Specifically, investigators first define the ideal experiment they would run if feasible and ethical. Such an experiment, in turn, identifies who enters the study (eligibility criteria), which policies are compared, when follow-up begins and ends, and which contrast of interventions defines the causal estimand. Once this ideal is stated, investigators  next arrange and check their observational data using methods that adjust for potential common causes of the exposure variable and outcomes of interest, and that adjust for loss to follow-up. Because the assumptions required to emulate an idealised experiment with observational data cannot generally be validated, investigators are encouraged to use sensitivity analyses [25].

Here, we implement target-trial emulation using modified treatment policies [26,27], which shift each person’s observed exposure from its observed value; we contrast the population projection of these shifts with the observed course (identity policy) [28–33]. This analytic strategy suits continuous exposures such as work hours. Using three annual waves of the New Zealand Attitudes and Values Study (NZAVS, *N* = 24,579) [34], we emulate that experiment. Time 12 (2020/2021) defines baseline (*T*_*0*_), Time 13 (2021/2022) is the exposure wave (*T*_*1*_), and Time 14 (2022/2023) is the outcome wave (*T*_*2*_). At *T*_*1*_, we shift weekly paid work hours by +10 or −10 and compare each policy with the observed course corrected for attrition. At *T*_*2*_, we measure 28 well-being indicators spanning psychological, physical, and social domains. This outcome-wide design avoids collapsing the analysis onto a single favourable Result [35,36].

## Method

We describe our target-trial emulation in the order of the trial we aim to approximate: target population, time zero (when eligibility is assessed and follow-up begins), intervention strategies, assignment, follow-up, outcomes, causal contrasts, and estimation. The eligible cohort comprised 24,579 adults from the NZAVS baseline wave who met the pre-retirement age restriction and had the required baseline measurements. The intervention occurs at *T*_*1*_, we measure outcomes at *T*_*2*_, and we define the two primary causal estimands as the population-average differences in mean well-being under the + 10-hour and −10-hour policies, each relative to the observed course. We estimate these contrasts with modified treatment policy estimators from the lmtp R package, using machine-learning adjustment for confounding and censoring [28,37]. We report the study following the TARGET (Transparent Reporting of Observational Studies Emulating a Target Trial) statement [24]; Supplement S8 provides the completed checklist S1 File.

### Methods at a glance

**Who entered the emulation?** 24,579 pre-retirement adults (aged 18–60) from the NZAVS 2020/2021 baseline with observed work hours, employment status, and survey weights.

**What did we intervene on?** Weekly hours of paid work at *T*_*1*_ (2021/2022).

**Which policies did we compare?** A + 10-hour policy, a −10-hour policy, and the observed course (censoring-adjusted) as the common reference condition.

**When did follow-up occur?** Baseline at *T*_*0*_ (2020/2021), intervention at *T*_*1*_ (2021/2022), outcomes measured at *T*_*2*_ (2022/2023).

**What did we estimate?** Two primary causal contrasts for the baseline cohort: + 10 versus observed course and −10 versus observed course, each across 28 well-being outcomes.

**How did we estimate it?** Modified treatment policy estimation, implemented with the lmtp R package and machine-learning models for exposure, censoring, and outcomes [28,37].

### Sample

The New Zealand Attitudes and Values Study is an ongoing nationwide longitudinal (panel) study that has collected annual data from New Zealand residents since 2009 [34]. Participants complete the questionnaire by mail or online and provide written informed consent. After each wave, the study offers participants entry into a prize draw to win one of five NZ$1000 grocery vouchers. The University of Auckland Human Participants Ethics Committee granted ethics approval (reference 014889, 2015–2021; UAHPEC22576, 2021–2027). We accessed the baseline-to-outcome data on 27/06/2024. We did not preregister the study because the ongoing panel had already collected the data.

The target population is pre-retirement adults in New Zealand aged 18–60 at baseline, regardless of employment status. We adopted this age restriction to reduce conflation of ordinary work-hour changes with retirement transitions. To obtain valid inference for this population, we incorporated New Zealand 2018 census survey weights for age, gender, and ethnicity [34], because these weights provide better coverage than the older alternative based on European ethnicity.

We assessed eligibility at baseline: participants had to be aged 18–60 and have observed baseline work hours, employment status, and survey weights. We assigned the work-hours policy at the exposure wave and treated later non-response as censoring rather than as a condition for study entry.

Under this eligibility rule, 24,579 participants entered the cohort at baseline. Of these, 17,634 reported work hours at the exposure wave and 15,275 were observed at the outcome wave. Supplement S1 reports baseline descriptive summaries and exposure distributions across waves, and Supplement S3 reports the eligibility audit and wave support (S1 File).

### Target trial

The emulated trial begins with the eligible baseline cohort described above. Eligibility and time zero both occur at *T*_*0*_. This alignment ensures that we measure covariates before the intervention and avoid conditioning study entry on later observation. The data contain no observed experimental groups. Instead, we define the + 10-hour, observed-course, and −10-hour arms as counterfactual projections of that same cohort under alternative work-hour policies.

We apply the intervention once at *T*_*1*_. Under the **+ 10-hour policy**, each person’s observed weekly paid work hours increase by 10. Under the **−10-hour policy**, each person’s observed weekly paid work hours decrease by 10, with a floor at zero. Under the **identity policy**, work hours remain as observed. Combined with a no-censoring intervention, the identity policy defines the observed course corrected for attrition. The two primary causal estimands are therefore the population-average differences in mean outcome between each shift policy and the observed course.

Using the observed course as the common reference serves two purposes. Substantively, the effects of increased work hours need not mirror the effects of decreased work hours, so a common reference lets us assess asymmetry on the same scale. Empirically, the reference arm stays closer to the data: its exposure history is the one participants actually reported, so we need to model only the no-censoring component. A design that compared the two shifted policies directly would require empirical support for hypothetical exposure histories in both arms, placing a stronger demand on positivity [28]. Supplement S4 gives the formal policy definitions (S1 File).

**Fig 2:**
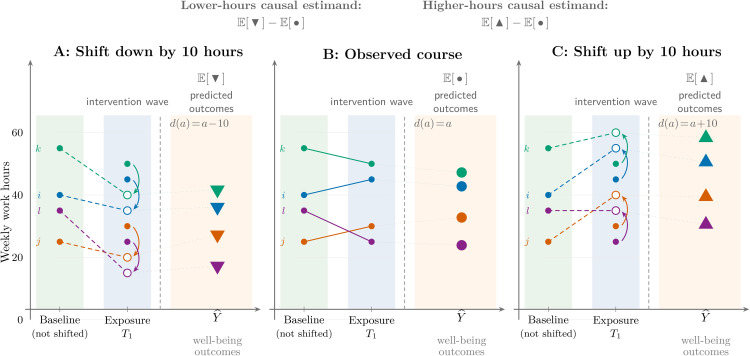
Detailed illustration of the emulated target trial. Panel B shows the observed-course identity policy (*d*(*a*) = ***a*)**. Panels A and C show the two shifted policies (d(a)=a−10 and *d*(*a*) = *a* + 10), which move each person’s observed *T*_*1*_ work hours down or up by 10 hours. Filled circles mark observed values, open circles mark policy-modified exposures, and dashed lines trace the counterfactual path to *T*_*1*_. Downward triangles mark predicted outcomes under the −10 policy (Panel **A)**, filled circles mark predicted outcomes under the observed course (Panel **B)**, and upward triangles mark predicted outcomes under the + 10 policy (Panel **C)**. Symbols match Fig 1. The causal estimand annotations at the top show the two contrasts. We condition on baseline variables rather than intervene on them, and we treat loss before *T*_*1*_ or *T*_*2*_ as censoring.

Fig 2 summarises the emulated trial. Panel B serves as the reference condition, in which work hours at *T*_*1*_ remain as observed and we estimate outcomes at *T*_*2*_. Panels A and C show the two shifted policies, which move each person’s observed *T*_*1*_ work hours down or up by 10 hours. We condition on baseline variables rather than intervene on them, and we treat participants lost before *T*_*1*_ or *T*_*2*_ as censored. Inverse probability of censoring weights then reweight the observed data so that estimation targets the mean outcome that would have been observed in the baseline cohort under each policy had censoring not occurred.

### Exposure and outcomes

The New Zealand Attitudes and Values Study assesses weekly hours of paid work using an open-ended item asking participants to estimate time spent in paid employment in the previous week. Because the item refers only to the previous week, it is vulnerable to random week-to-week noise from holidays, illness, or unusually high or low workloads. It is also an incomplete measure of labour-market history. A person moving from 50 to 60 hours after years of long-hour work may differ from a person newly moving from 20 to 30 hours, even if both receive the same +10-hour shift. Such exposure heterogeneity affects interpretation, and largely uncorrelated week-to-week measurement error would ordinarily attenuate both associational and causal estimates towards zero. We evaluated 28 well-being outcomes spanning five domains (Table 1). Supplement S2 details the outcome measures, and Supplement S1 reports baseline demographics, exposure distributions, and outcome distributions (S1 File).

**Table 1:** Well-being outcomes by domain.

Domain	Indicators
Present-focussed well-being	Perceived self-control, desire for more self-control, self-esteem, perfectionism
Embodied well-being	Body satisfaction, Kessler-6 anxiety, Kessler-6 depression, sexual satisfaction, fatigue, rumination
Life-focussed well-being	Personal Well-Being (PWB) satisfaction with standard of living, health, relationships, and future security; meaning in life, life satisfaction, sense of purpose, vengefulness, gratitude
Biological health	Short-form subjective health, hours of sleep, hours of exercise, alcohol intensity, alcohol frequency, BMI
Social well-being	Social belonging, social support, neighbourhood community

The design fixes temporal ordering and addresses reverse causation by ensuring that we measure all conditioning variables before the intervention. We measure baseline covariates, baseline work hours, and baseline values of all outcomes at *T*_*0*_. We intervene on work hours at *T*_*1*_. We measure outcomes at *T*_*2*_.

We adopt VanderWeele’s outcome-wide approach, which simultaneously examines multiple outcomes rather than selecting a single measure of well-being [35]. This design reduces outcome selection and keeps the analysis open to the possibility that work-hour changes affect some domains but not others.

**Fig 3:**
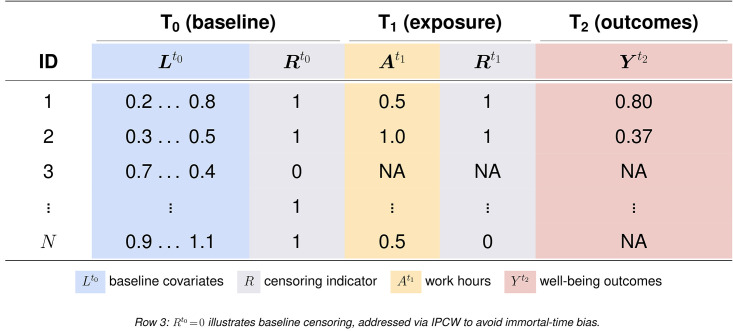
Data structure used in the modified treatment policy analysis, implemented with lmtp (adapted from [38]). Each row is one participant. We measure baseline covariates ($L^{t_{0}}$) [which include baseline exposure ($A^{t_{0}}$) and baseline outcomes ($Y^{t_{0}}$)] before the exposure at *T*_*1*_. Each *R* column indicates observation status at the subsequent wave. We handle censoring with inverse probability of censoring weights [39], so the analysis targets the baseline cohort under each policy had censoring not occurred.

Fig 3 shows the data passed to the modified treatment policy estimator. Each row represents one participant in the baseline-defined cohort. The baseline block contains baseline covariates, baseline work hours, and baseline values of all outcomes. The exposure at *T*_*1*_ is weekly work hours, and the outcomes at *T*_*2*_ are the 28 standardised well-being measures. Censoring indicators record whether each participant remains observed at the next wave. Because loss to follow-up can depend on prior history, we model censoring and use inverse probability of censoring weights so that the causal estimand remains the mean outcome that would have been observed in the baseline cohort under each policy had censoring not occurred.

### Confounding control

The causal interpretation rests on three assumptions. **Consistency** requires the observed outcome under the work-hour value actually received to equal the corresponding counterfactual outcome. This assumption also requires the work-hour shift to be coherent enough for interpretation. A 10-hour increase can reflect a promotion, voluntary overtime, mandatory overtime, understaffing, business growth, or financial strain. These different versions may have different consequences [40–42]. The estimated contrast can remain well defined as an average over versions present in the study population, but the policy interpretation becomes less precise when those versions have heterogeneous effects. **Conditional exchangeability** requires that, after adjustment for measured baseline history, the shifted and reference exposure values are as if randomly assigned. This assumption is untestable in observational data. **Positivity** requires that people with similar measured histories still show a range of work-hour values in the data; we refer to this empirical side of positivity as “overlap”. Supplement S3 (S1 File) formalises all three assumptions and the overlap checks for the positivity assumption; for accessible introductions to causal inference and its assumptions see [43] and [44].

**Fig 4:**
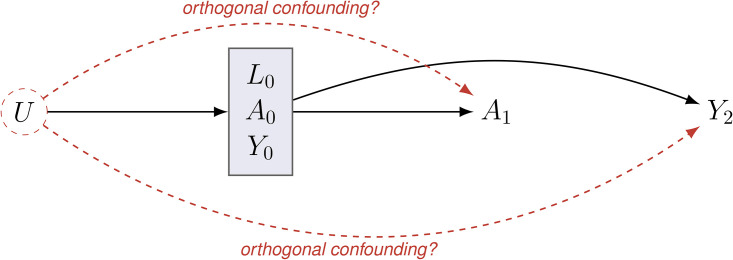
Confounding-control strategy as a causal directed acyclic graph (causal DAG). We condition on the baseline block (*L*_0_, *A*_0_, *Y*_0_) (boxed). Solid arrows represent structural paths blocked by this conditioning. Dashed red arrows represent potential orthogonal confounding: unmeasured factors (*U*) that could open paths between *A*_1_ and *Y*_2_ through mechanisms not captured by the measured baseline history. We use the causal DAG to assess whether conditioning on the baseline block blocks all non-causal paths between the exposure and outcome [43].

We selected adjustment variables using VanderWeele’s modified disjunctive cause criterion, which includes any variable that causes the exposure, the outcome, or both [45] (see Supplement S2 for the full list and Supplement S1 for baseline demographics (S1 File)). We adjusted for baseline covariates that are common causes of work hours and well-being, including demographic, socioeconomic, personality, health, employment, and time-use variables. The adjustment set also included baseline work hours and baseline values of all outcomes, as well as the baseline value of the exposure. We did not adjust for variables measured at or after the exposure wave, because conditioning on post-exposure variables can introduce mediator bias [46]. Fig 4 represents our confounding control strategy using a causal directed acyclic graph (causal DAG) [43].

Note that in our study, any remaining unmeasured confounder would need to affect both work hours in the exposure wave and later well-being outcomes measured at the end of study independently of all baseline covariates, a set which includes the baseline measure of work hours and the baseline measures of all outcome variables. Baseline covariate adjustment does not block confounders that change between *T*_*0*_ and *T*_*1*_, such as a health problem arising after the baseline wave. Hence, as a sensitivity analysis, we report *E*-values, which state the minimum strength of association an unmeasured confounder would need with both the exposure and the outcome, above and beyond the measured covariates, to explain away the observed effect (Supplement S6 in S1 File) [47].

We separately handled attrition as potentially informative censoring. Restricting. the analysis to participants observed at all later waves would change the target population from the eligible baseline cohort to a retained complete-case subset. That restriction could introduce selection bias. We therefore modelled non-response between waves with wave-specific censoring indicators and used inverse probability of censoring weights to recover the mean outcome that would have been observed in the baseline cohort under each policy had censoring not occurred. This approach assumes that censoring is conditionally random given the measured baseline history.

### Missing data

We handled missing data in two stages. First, we imputed missing baseline covariates and baseline outcome values using predictive mean matching [48]. Second, as mentioned, we used inverse probability of censoring weights to target the baseline cohort under complete follow-up. This strategy relies on missing-at-random assumptions for baseline imputation and censoring-at-random assumptions for post-baseline non-observation. Supplement S3 gives further information about our missing data handling strategies (S1 File).

### Estimation

Because the exposure is continuous and the policies shift observed work hours locally, practical positivity is a design requirement: the observed data must contain adequate support for the policy-modified exposure values within levels of measured history. In preliminary analyses, the originally planned ±20-hour shifts moved too many participants into weakly supported regions of the exposure distribution, so we refined the causal estimand to the better-supported ±10-hour policies. We report positivity diagnostics in the Results and Supplement S5 (S1 File).

We estimated policy effects using modified treatment policy estimators implemented in the lmtp R package [28,37]. The estimator fits models for the exposure process, the censoring process, and the outcome, then combines them so that the estimate remains consistent if either the outcome model is correctly specified or both the exposure and censoring models are correctly specified (the doubly robust property) [29,38,49,50]. To reduce overfitting, we used five-fold cross-fitting: we split the data into five subsets, estimated the exposure, censoring, and outcome models for each subset using models trained on the remaining four subsets, and averaged the final estimate across all five splits. This procedure ensures that no observation’s outcome influences the model used to predict it.

We conditioned on baseline work hours and applied the intervention only at the exposure wave. lmtp internally computed the reference condition as the policy that leaves hours as observed, so the reported contrasts compare each shift-policy mean with the observed-course mean and target the baseline cohort under complete follow-up. We right-tail trimmed census weights at the 99th percentile and passed them to lmtp as baseline weights.

We standardised all 28 outcomes to z-scores (mean zero, unit variance) so that the estimated contrasts are directly interpretable as effect sizes in standard-deviation units. We estimated three model components with five-fold cross-fitted Super Learner ensembles: the exposure model, the censoring model, and the outcome model. Super Learner combines several machine-learning methods (a simple mean, random forests, gradient boosting, and regularised regression) and selects the weighted combination that predicts best [50]. The baseline adjustment set comprised the selected covariates, baseline work hours, and baseline values of all outcomes. We estimated population-average effects only; we did not estimate subgroup-specific effects because the combination of 28 outcomes with multiple subgroups would raise multiplicity and positivity challenges.

Because we evaluated 28 outcomes under each policy, we applied Bonferroni correction, which divides the conventional significance threshold by the number of outcomes (α/28) to control the probability of any false positive, yielding 99.82% confidence intervals. We recomputed *E*-values from the corrected intervals [25,46]. We report *E*-values descriptively rather than applying a hard threshold. Modest *E*-values indicate greater vulnerability to residual confounding, but measurement error in the single-week work-hours item likely attenuates both effect estimates and their *E*-values, so the reported values are conservative. Supplement S3 expands the missing-data and cohort-support details, Supplement S5 reports the positivity diagnostics, and Supplement S6 reports the full numerical contrasts on both scales (S1 File).

## Results

**Fig 5:**
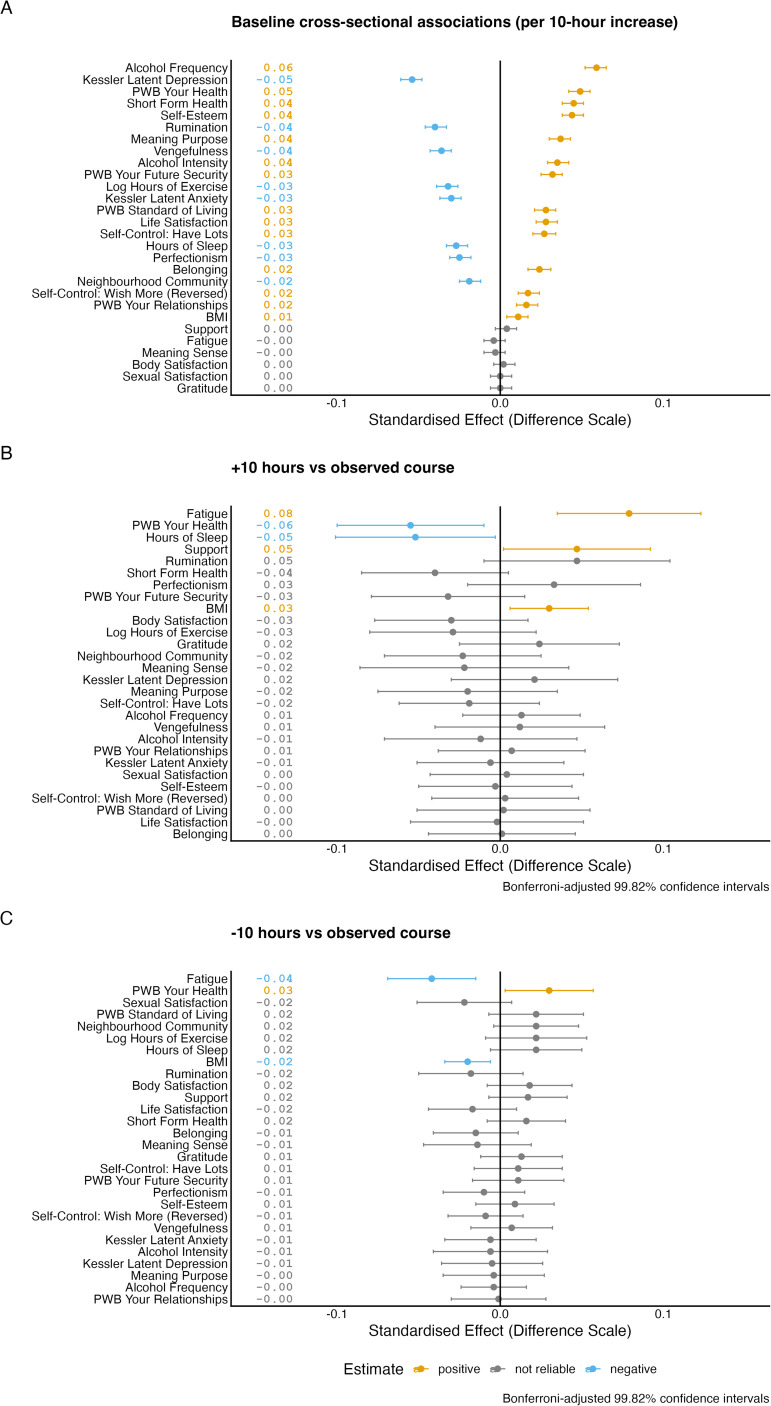
Panels compare (A) descriptive baseline cross-sectional associations between baseline work hours and baseline well-being outcomes in the eligible cohort, which suggest a broader and sometimes contradictory pattern because they use only cross-sectional data and omit confounding control, (B) causal effect sizes for shifting weekly work hours +10 compared with the observed course, and (C) causal effect sizes for shifting weekly work hours −10 compared with the observed course. Panels B and C are population-average causal contrasts under the stated identification assumptions. All estimates are in standardised units. Orange indicates a positive effect, blue a negative effect, and grey an effect whose corrected confidence interval includes the null.

We report results on two scales. The standardised (z-transformed) scale expresses each contrast in standard-deviation units, making the 28 outcomes directly comparable. The original scale converts those same contrasts back into the outcome’s natural units by multiplying the z-scale estimate by the outcome-wave standard deviation. For item-based measures, we report the original questionnaire response scale; for sleep, we report hours; and for BMI, we report kg/m^2^. The baseline cross-sectional coefficients in Panel A of Fig 5 remain descriptive associations within the eligible baseline cohort on the standardised scale. Panel A is interesting because it indicates what the data would suggest about a 10-hour difference in work hours if we were to rely only on cross-sectional data and omit confounding control.

### Positivity and empirical support (± 10-hour causal estimands)

The lmtp estimator reweights observed person-time so that the weighted data approximate a population in which everyone’s work hours follow the specified policy. When a shifted exposure value is rare given a person’s history, the corresponding density ratio becomes small, so that person contributes little direct information to the estimate. Ratios near 1 mean the shifted exposure is well supported by the data; ratios near 0 or much greater than 1 mean the estimate relies more on model extrapolation than on direct observation. A large fraction of near-zero weight-products therefore signals greater reliance on extrapolation rather than direct empirical support.

For the ± 10-hour policies, overlap improved relative to the originally planned ± 20-hour shifts but remained asymmetric. Under the + 10-hour policy, 76.8% of uncensored rows remained within the product-of-ratio interval [0.1, 10], while 20.0% fell below 0.1 and 3.3% exceeded 10, indicating weaker support for the upward-shift intervention. Under the −10-hour policy, support was much stronger: 96.2% of uncensored rows remained within [0.1, 10], and only 3.8% fell below 0.1. The observed-course identity policy showed no corresponding support strain.

The effective sample-size summaries point in the same direction. After trimming, cumulative effective sample size (ESS) was approximately 2,110 for the + 10-hour policy, 4,123 for the −10-hour policy, and 9,922 for the observed-course identity policy in the baseline-defined eligible cohort (*N* = 24,579). Thus, although the + 10 contrast remained estimable, it relied on a materially narrower region of support than the −10 contrast and warrants more cautious interpretation.

### Comparison of descriptive and causal estimates

Fig 5 compares all three sets of estimates on the same standardised effect scale. Panel A shows descriptive baseline cross-sectional associations between baseline work hours and baseline well-being in the eligible cohort. Panels B and C report population-average causal effect estimates for the same baseline cohort under the + 10 and −10 policies relative to the observed course (censoring-adjusted). In Panel A, many coefficients exclude zero and some even look beneficial, including higher self-esteem and lower anxiety, yet the same descriptive model shows almost no association with fatigue or support, only a slight positive slope for BMI, and better perceived health. Once we impose temporal ordering, adjust for baseline outcomes and covariates, and weight responses for attrition, the pattern considerably  narrows, several coefficients change sign, and fatigue, perceived health, and BMI emerge as causal effects. The full descriptive table for all results reported in Fig 5 appears in Supplement S7 (S1 File).

### Shift +10 hours of work per week

We report estimated average treatment effects (ATE) for each contrast. To assess sensitivity to unmeasured confounding, we computed *E*-values [47]. An *E*-value gives the minimum strength of association that an unmeasured confounder would need with both work hours and the outcome, above and beyond the measured covariates, to explain away the observed effect.

After Bonferroni correction for 28 outcomes, the effect least vulnerable to unmeasured confounding under the + 10-hour policy is higher fatigue (ATE = 0.079, corrected CI = [0.035, 0.123], *E*-value bound = 1.22), equivalent to about 0.09 points on the original fatigue scale. Explaining away this result would require an unmeasured confounder associated with both work hours and fatigue by at least 1.22 beyond the measured baseline history. Lower perceived health on the PWB health item (ATE = −0.055, corrected CI = [−0.100, −0.010], *E*-value bound = 1.10) corresponds to a decline of about 0.13 points on the original item scale. Several other effects are directionally adverse, including fewer hours of sleep (ATE = −0.052), or about 3.4 fewer minutes, lower short-form health (ATE = −0.040), or about −0.05 points, and higher BMI (ATE = 0.030), or about +0.19 kg/m^2^, but their *E*-value bounds are more modest or their corrected confidence intervals include zero. One small countervailing shift is greater perceived support (ATE = 0.047, corrected CI = [0.002, 0.092]), equivalent to about +0.06 points on the original scale, but its *E*-value bound is only 1.04, so we treat it cautiously. The adverse pattern is concentrated in recovery and embodied health rather than spread across all 28 outcomes. Supplement S6 reports full outcome tables on both scales (S1 File).

### Shift −10 hours of work per week

Under the −10-hour policy, the effect least vulnerable to unmeasured confounding after Bonferroni correction is lower fatigue (ATE = −0.042, corrected CI = [−0.069, −0.015], *E*-value bound = 1.13), equivalent to about 0.05 points on the original fatigue scale. Lower BMI (ATE = −0.020, corrected CI = [−0.034, −0.006]) corresponds to about −0.13 kg/m^2^, and higher perceived health on the PWB health item (ATE = 0.030, corrected CI = [0.003, 0.057]) corresponds to about +0.07 points on the original item scale. Both retain corrected intervals that exclude zero, but their *E*-value bounds (1.08 and 1.06) are modest. Several other shifts are directionally favourable, including more sleep, more exercise, and greater body satisfaction, but they do not survive correction. The beneficial pattern is again concentrated in a few domains rather than spread across all outcomes. Supplement S6 reports full outcome tables on both scales (S1 File).

## Discussion

Under the stated identification assumptions, work-hour shifts affect a narrow cluster of recovery and health outcomes more clearly than well-being as a whole. At baseline, longer hours correlate with differences across many domains, some apparently beneficial. Once we fix eligibility, shift hours at *T*_*1*_, adjust for baseline covariates (including baseline work hours and all baseline outcomes), and weight for attrition, most associations shrink markedly and many corrected intervals include zero.

Panel A of Fig 5 shows that most baseline coefficients in the cross-sectional model (Panel A) exclude zero. At the same time, this descriptive model shows almost no association for hours of work with fatigue or support, and only a slight positive slope for BMI -- associations that appear as causal effects in the longitudinal modified treatment policy models (Fig 5, Panels B and C). Taken together, these findings imply that cross-sectional associations may be extremely misleading as guides to causality. The sign reversals are especially instructive. At baseline, longer work hours are descriptively associated with better perceived health, better short-form health, higher self-esteem, more meaning, and less rumination and depressive symptomatology. Under the emulated +10-hour policy, the corresponding causal estimates show no such detectable benefit, or show adverse effects: more fatigue, less sleep, worse perceived health, and higher BMI. The recovery-and-health cluster is the most internally coherent pattern in the longitudinal modified treatment policies, both for the gain and loss interventions. When work hours increase by 10, fatigue rises by about 0.09 points, sleep falls by about 0.06 hours, perceived health worsens by about 0.13 points, and BMI increases by about 0.19 kg/m^2^. When hours decrease by 10, fatigue falls by about 0.05 points, BMI falls by about 0.13 kg/m^2^, and perceived health improves by about 0.07 points. A small increase in perceived support under the + 10-hour policy may reflect the social side of work, but its resistance to unmeasured confounding is weak, so we treat this finding as provisional. Although these results indicate modest effect sizes, we should not rule them out as trivial. In epidemiology, small shifts in exposure matter when they move the distribution of outcomes across a whole population [51,52]. The clearest surviving effects cluster around recovery and embodied strain, and the surrounding point estimates moreover tilt in the same direction. Even modest shifts in fatigue, sleep, or BMI could have public health implications operating over a population at scale.

### Limitations

First, uncertainty  remains substantial. Many point estimates attenuate towards zero after confounding control and censoring adjustment, and many Bonferroni-corrected intervals include zero. Failure to exclude effects does not confirm a null.

Second, results are defined for a specific comparison of shift interventions in a single population. Results might not generalise to comparisons of different shift interventions or to other populations.

Third, any causal interpretation of our results rests on assumptions. Exchangeability requires no unmeasured common causes of work-hour changes and later well-being after conditioning on baseline history. As mentioned, this assumption cannot be verified, and we rely on interpreting E-values for sensitivity analysis. Furthermore, even if the 10-hour shift it is not readily interpretable across the many versions of treatment estimated in this study, as people gain and lose work for a variety of reasons, such as required overtime, promotion, demotion, caregiving, financial hardship, inheritance, or any number of unmeasured health issues [41,42]. Our estimates are obtained from the distribution of these many treatment versions. Reducing work hours because one has had a demotion, say, is likely to differ in its effects on well-being compared with reducing work hours because one has inherited a fortune.

Fourth, participants self-report both work hours and well-being outcomes. If measurement errors in these reports are distributed randomly, they will likely attenuate true effects [53–55].

## Contribution and Further Direction

Even considering the limitations just described, the present design contributes something that the usual work-hours literature often lacks. The target-trial framework forces investigators to state the intervention, the target population, the start of follow-up, the comparison condition, and the identifying assumptions before statistical estimation begins. The outcome-wide design prevents the analysis from collapsing onto a single favourable measure. Here, a causal framework combines with a large national longitudinal (panel) study that affords rich baseline history and repeated measurement to align data with causality’s temporal arrow [34]. Recent applications already show that target-trial emulation using observational data can recover the results of randomised controlled epidemiological experiments [30–33]; the current study extends the target trial framework to the study of work hours and well-being, an area for which randomised controlled studies are inevitably limited by costs and ethics. Furthermore, we believe the policy implications of our findings are practically important. Even a modest reduction of ten hours per week points to measurable benefits for fatigue, perceived health, and BMI. That places recovery-protective policies on firmer ground, including caps on weekly hours, right-to-disconnect legislation, and protected rest periods. Such policies should nonetheless be advanced cautiously, given the limitations just described and because we do not yet understand for whom the benefits of work reduction are largest, or who may not benefit at all -- fundamental questions for future research [56].

## Supporting information

S1 File Supplement S1 reports descriptive statistics for the baseline covariates, exposure distribution, and outcome distributions. Supplement S2 provides measure descriptions. Supplement S3 expands the identification assumptions, missing-data strategy, and cohort-support audit. Supplement S4 gives the formal policy definitions and censoring intervention. Supplement S5 reports positivity diagnostics. Supplement S6 reports the primary modified treatment policy effect tables on both the standardised and original scales. Supplement S7 reports the descriptive baseline cross-sectional associations. Supplement S8 provides the TARGET checklist.
(PDF)
