# Supplementary material for: How work hours affect well-being: A target trial emulation
Source: PLoS One. 2026 Jun 8;21(6):e0350816. doi: 10.1371/journal.pone.0350816 (PMC13245862; doi:10.1371/journal.pone.0350816)
Supplement: S1 File — Supplement S2 provides measure descriptions. Supplement S3 expands the identification assumptions, missing-data strategy, and cohort-support audit. Supplement S4 gives the formal policy definitions and censoring intervention. Supplement S5 reports positivity diagnostics. Supplement S6 reports the primary modified treatment policy effect tables on both the standardised and original scales. Supplement S7 reports the descriptive baseline cross-sectional associations. Supplement S8 provides the TARGET checklist. (PDF) [file pone.0350816.s001.pdf]

Supplementary Materials: How Work Hours Affect Well-being: A Target Trial Emulation

Anonymous

These supplementary materials provide descriptive tables, measure descriptions, the identification and missing-data rationale, formal policy definitions, positivity diagnostics, full numerical contrasts on the standardised and original scales, a naive associational comparison, and the TARGET checklist for the target-trial emulation of work-hour effects on well-being.

Supplement S1: Descriptive Tables

Table S1 summarises the baseline covariates used for confounding control across the eligible cohort.

Table 1. Table S1. Baseline covariates in the eligible cohort.

	T0 (2020/2021)
	(N=24579)
**Age**	
Mean (SD)	45.8 (10.9)
Median [Min, Max]	48.0 [18.0, 60.0]
**Agreeableness**	
Mean (SD)	5.37 (0.995)
Median [Min, Max]	5.50 [1.00, 7.00]
Missing	138 (0.6%)
**Social Belonging**	
Mean (SD)	4.94 (1.13)
Median [Min, Max]	5.00 [1.00, 7.00]
Missing	144 (0.6%)
**Born in NZ**	
0	5309 (21.6%)
1	19255 (78.3%)
Missing	15 (0.1%)
**Charitable Donations (log)**	
Mean (SD)	4.41 (2.73)
Median [Min, Max]	5.02 [0, 12.4]
Missing	263 (1.1%)
**Conscientiousness**	
Mean (SD)	5.07 (1.07)
Median [Min, Max]	5.25 [1.00, 7.00]
Missing	138 (0.6%)
**Education Level (0-10)**	
no_qualification	318 (1.3%)
cert_1_to_4	6235 (25.4%)
cert_5_to_6	2954 (12.0%)
university	7544 (30.7%)
post_grad	3762 (15.3%)
masters	2830 (11.5%)
doctorate	825 (3.4%)

	T0 (2020/2021)
Missing	111 (0.5%)
**Employed**	
0	2780 (11.3%)
1	21799 (88.7%)
**Ethnicity**	
euro	19619 (79.8%)
maori	2968 (12.1%)
pacific	591 (2.4%)
asian	1227 (5.0%)
Missing	174 (0.7%)
**Extraversion**	
Mean (SD)	3.85 (1.23)
Median [Min, Max]	3.75 [1.00, 7.00]
Missing	137 (0.6%)
**Disability**	
0	18636 (75.8%)
1	5785 (23.5%)
Missing	158 (0.6%)
**Honesty-Humility**	
Mean (SD)	5.48 (1.14)
Median [Min, Max]	5.75 [1.00, 7.00]
Missing	143 (0.6%)
**Hours of Charity (log)**	
Mean (SD)	0.345 (0.712)
Median [Min, Max]	0 [0, 4.80]
**Hours with Children (log)**	
Mean (SD)	1.24 (1.68)
Median [Min, Max]	0 [0, 5.13]
**Hours Commuting (log)**	
Mean (SD)	1.40 (0.832)
Median [Min, Max]	1.39 [0, 4.62]
**Hours of Exercise (log)**	
Mean (SD)	1.54 (0.822)
Median [Min, Max]	1.61 [0, 4.39]
**Hours of Housework (log)**	
Mean (SD)	2.20 (0.719)
Median [Min, Max]	2.40 [0, 5.13]
**Household Income (log)**	
Mean (SD)	11.6 (0.796)
Median [Min, Max]	11.7 [0.693, 14.9]
Missing	582 (2.4%)
**Kessler Latent Anxiety**	
Mean (SD)	1.31 (0.765)
Median [Min, Max]	1.33 [0, 4.00]
Missing	140 (0.6%)
**Kessler Latent Depression**	
Mean (SD)	0.637 (0.769)
Median [Min, Max]	0.333 [0, 4.00]
Missing	139 (0.6%)
**Male**	
0	16280 (66.2%)

	T0 (2020/2021)
1	8118 (33.0%)
Missing	181 (0.7%)
**Neuroticism**	
Mean (SD)	3.62 (1.17)
Median [Min, Max]	3.50 [1.00, 7.00]
Missing	139 (0.6%)
**NZ Deprivation Index**	
Mean (SD)	4.67 (2.73)
Median [Min, Max]	4.00 [1.00, 10.0]
Missing	300 (1.2%)
**Socioeconomic Index (NZSEI)**	
Mean (SD)	56.6 (16.7)
Median [Min, Max]	61.0 [10.0, 90.0]
Missing	79 (0.3%)
**Openness**	
Mean (SD)	5.05 (1.12)
Median [Min, Max]	5.00 [1.00, 7.00]
Missing	139 (0.6%)
**Parent**	
0	7910 (32.2%)
1	16669 (67.8%)
**Partner**	
0	5844 (23.8%)
1	18583 (75.6%)
Missing	152 (0.6%)
**Political Conservatism**	
Mean (SD)	3.30 (1.35)
Median [Min, Max]	3.00 [1.00, 7.00]
Missing	516 (2.1%)
**Religious Identification**	
Mean (SD)	2.18 (2.09)
Median [Min, Max]	1.00 [1.00, 7.00]
Missing	128 (0.5%)
**Rural (GCH 2018)**	
1	15514 (63.1%)
2	4470 (18.2%)
3	2848 (11.6%)
4	1208 (4.9%)
5	270 (1.1%)
Missing	269 (1.1%)
**Sample Frame Opt-In**	
0	21768 (88.6%)
1	2811 (11.4%)
**Sample Origin**	
1-2	1125 (4.6%)
3-3.5	847 (3.4%)
4	1266 (5.2%)
5-6-7	2723 (11.1%)
8-9	3243 (13.2%)
10	12920 (52.6%)
11 and above	2455 (10.0%)

	T0 (2020/2021)
**Short Form Health**	
Mean (SD)	5.01 (1.16)
Median [Min, Max]	5.00 [1.00, 7.00]
**Social Support**	
Mean (SD)	5.91 (1.16)
Median [Min, Max]	6.33 [1.00, 7.00]
Missing	10 (0.0%)

Table S2 reports the work-hours distribution at baseline and at the exposure wave.

Table 2. Table S2. Work-hours distribution at baseline and the exposure wave.

	T0 (2020/2021)	T1 (2021/2022)
	(N=24579)	(N=24579)
**Hours of Work Per Week**		
Mean (SD)	29.9 (19.1)	30.5 (18.4)
Median [Min, Max]	38.0 [0, 168]	37.5 [0, 113]
Missing	0 (0%)	7474 (30.4%)

Table S3 reports descriptive statistics for the 28 well-being outcomes at baseline and at the outcome wave.

Table 3. Table S3. Well-being outcomes at baseline and the outcome wave.

	T0 (2020/2021)	T2 (2022/2023)
	(N=24579)	(N=24579)
**Alcohol Frequency**		
Mean (SD)	2.11 (1.31)	2.03 (1.32)
Median [Min, Max]	2.00 [0, 4.00]	2.00 [0, 4.00]
Missing	292 (1.2%)	9399 (38.2%)
**Alcohol Intensity**		
Mean (SD)	2.12 (2.07)	1.98 (1.89)
Median [Min, Max]	2.00 [0, 36.0]	2.00 [0, 40.0]
Missing	739 (3.0%)	9808 (39.9%)
**Social Belonging**		
Mean (SD)	4.94 (1.13)	5.04 (1.14)
Median [Min, Max]	5.00 [1.00, 7.00]	5.00 [1.00, 7.00]
Missing	144 (0.6%)	9378 (38.2%)
**Body Satisfaction**		
Mean (SD)	4.03 (1.70)	4.05 (1.71)
Median [Min, Max]	4.00 [1.00, 7.00]	4.00 [1.00, 7.00]
Missing	181 (0.7%)	9934 (40.4%)
**Gratitude**		
Mean (SD)	5.95 (0.875)	5.93 (0.909)
Median [Min, Max]	6.00 [1.00, 7.00]	6.00 [1.00, 7.00]
Missing	5 (0.0%)	9349 (38.0%)
**BMI**		
Mean (SD)	27.6 (6.12)	27.8 (6.27)
Median [Min, Max]	26.3 [12.5, 70.9]	26.5 [13.9, 70.9]
Missing	209 (0.9%)	9375 (38.1%)

	T0 (2020/2021)	T2 (2022/2023)
**Fatigue**		
Mean (SD)	1.82 (1.07)	1.83 (1.08)
Median [Min, Max]	2.00 [0, 4.00]	2.00 [0, 4.00]
Missing	208 (0.8%)	9376 (38.1%)
**Hours of Sleep**		
Mean (SD)	6.91 (1.09)	6.92 (1.09)
Median [Min, Max]	7.00 [2.00, 15.0]	7.00 [2.00, 14.0]
Missing	636 (2.6%)	9925 (40.4%)
**Hours of Exercise (log)**		
Mean (SD)	1.54 (0.822)	1.56 (0.811)
Median [Min, Max]	1.61 [0, 4.39]	1.61 [0, 4.39]
Missing	0 (0%)	9770 (39.7%)
**Kessler Latent Anxiety**		
Mean (SD)	1.31 (0.765)	1.30 (0.775)
Median [Min, Max]	1.33 [0, 4.00]	1.33 [0, 4.00]
Missing	140 (0.6%)	9342 (38.0%)
**Kessler Latent Depression**		
Mean (SD)	0.637 (0.769)	0.618 (0.768)
Median [Min, Max]	0.333 [0, 4.00]	0.333 [0, 4.00]
Missing	139 (0.6%)	9342 (38.0%)
**Life Satisfaction**		
Mean (SD)	5.12 (1.29)	5.09 (1.28)
Median [Min, Max]	5.50 [1.00, 7.00]	5.50 [1.00, 7.00]
Missing	300 (1.2%)	9719 (39.5%)
**Meaning: Purpose**		
Mean (SD)	5.00 (1.51)	5.05 (1.46)
Median [Min, Max]	5.00 [1.00, 7.00]	5.00 [1.00, 7.00]
Missing	361 (1.5%)	10169 (41.4%)
**Meaning: Sense**		
Mean (SD)	5.62 (1.25)	5.66 (1.25)
Median [Min, Max]	6.00 [1.00, 7.00]	6.00 [1.00, 7.00]
Missing	43 (0.2%)	9931 (40.4%)
**Neighbourhood Community**		
Mean (SD)	4.19 (1.61)	4.42 (1.59)
Median [Min, Max]	4.00 [1.00, 7.00]	5.00 [1.00, 7.00]
Missing	80 (0.3%)	9934 (40.4%)
**Perfectionism**		
Mean (SD)	3.21 (1.40)	3.15 (1.46)
Median [Min, Max]	3.00 [1.00, 7.00]	3.00 [1.00, 7.00]
Missing	1 (0.0%)	9410 (38.3%)
**PWB: Standard of Living**		
Mean (SD)	7.68 (1.96)	7.38 (2.13)
Median [Min, Max]	8.00 [0, 10.0]	8.00 [0, 10.0]
Missing	106 (0.4%)	9350 (38.0%)
**PWB: Future Security**		
Mean (SD)	6.26 (2.38)	5.87 (2.50)
Median [Min, Max]	7.00 [0, 10.0]	6.00 [0, 10.0]
Missing	50 (0.2%)	9370 (38.1%)
**PWB: Health**		
Mean (SD)	6.56 (2.32)	6.55 (2.40)
Median [Min, Max]	7.00 [0, 10.0]	7.00 [0, 10.0]

	T0 (2020/2021)	T2 (2022/2023)
Missing	78 (0.3%)	9384 (38.2%)
**PWB: Relationships**		
Mean (SD)	7.53 (2.31)	7.47 (2.31)
Median [Min, Max]	8.00 [0, 10.0]	8.00 [0, 10.0]
Missing	63 (0.3%)	9372 (38.1%)
**Rumination**		
Mean (SD)	0.917 (1.02)	0.887 (1.00)
Median [Min, Max]	1.00 [0, 4.00]	1.00 [0, 4.00]
Missing	181 (0.7%)	9379 (38.2%)
**Self-Control: Have Lots**		
Mean (SD)	4.79 (1.53)	4.91 (1.47)
Median [Min, Max]	5.00 [1.00, 7.00]	5.00 [1.00, 7.00]
Missing	72 (0.3%)	9996 (40.7%)
**Self-Control: Wish More (Reversed)**		
Mean (SD)	3.47 (1.77)	3.59 (1.79)
Median [Min, Max]	3.00 [1.00, 7.00]	3.00 [1.00, 7.00]
Missing	58 (0.2%)	9897 (40.3%)
**Self-Esteem**		
Mean (SD)	4.96 (1.34)	5.08 (1.35)
Median [Min, Max]	5.00 [1.00, 7.00]	5.33 [1.00, 7.00]
Missing	143 (0.6%)	9365 (38.1%)
**Sexual Satisfaction**		
Mean (SD)	4.39 (1.78)	4.34 (1.78)
Median [Min, Max]	5.00 [1.00, 7.00]	4.00 [1.00, 7.00]
Missing	707 (2.9%)	10053 (40.9%)
**Short Form Health**		
Mean (SD)	5.01 (1.16)	4.85 (1.18)
Median [Min, Max]	5.00 [1.00, 7.00]	5.00 [1.00, 7.00]
Missing	0 (0%)	9344 (38.0%)
**Social Support**		
Mean (SD)	5.91 (1.16)	5.94 (1.19)
Median [Min, Max]	6.33 [1.00, 7.00]	6.33 [1.00, 7.00]
Missing	10 (0.0%)	9368 (38.1%)
**Vengefulness**		
Mean (SD)	2.97 (1.29)	2.89 (1.29)
Median [Min, Max]	2.67 [1.00, 7.00]	2.67 [1.00, 7.00]
Missing	4 (0.0%)	9392 (38.2%)

Supplement S2: Measure Descriptions

The following sections describe the baseline covariates, exposure, and outcomes used in the analysis.

Baseline Covariates

Age

We asked participants' ages in an open-ended question ("What is your age?" or "What is your date of birth") [1].

Items:

- What is your date of birth?

Agreeableness

Mini-IPIP6 Agreeableness dimension: (i) I sympathize with others' feelings. (ii) I am not interested in other people's problems. (r) (iii) I feel others' emotions. (iv) I am not really interested in others. (r) [2].

Items:

- I sympathize with others' feelings.
- I am not interested in other people's problems.
- I feel others' emotions.
- I am not really interested in others (reversed).

Social Belonging

We assessed felt belongingness with three items adapted from the Sense of Belonging Instrument (Hagerty & Patusky, 1995): (1) "Know that people in my life accept and value me"; (2) "Feel like an outsider"; (3) "Know that people around me share my attitudes and beliefs". Participants responded on a scale from 1 (Very Inaccurate) to 7 (Very Accurate). The second item was reversely coded [3].

Items:

- Know that people in my life accept and value me.
- Feel like an outsider (reversed).
- Know that people around me share my attitudes and beliefs.

Born in NZ

Coded binary (1 = New Zealand; 0 = elsewhere.) [1].

Items:

- Where were you born? (please be specific, e.g., which town/city?)

Charitable Donations (log)

Numerical: open-ended response [4].

Items:

- How much money have you donated to charity in the last year?

Conscientiousness

Mini-IPIP6 Conscientiousness dimension: (i) I get chores done right away. (ii) I like order. (iii) I make a mess of things. (r) (iv) I often forget to put things back in their proper place. (r) [2].

Items:

- I get chores done right away.
- I like order.
- I make a mess of things.
- I often forget to put things back in their proper place.

Education Level (0-10)

We asked participants, 'What is your highest level of qualification?'. We coded participants' highest finished degree according to the New Zealand Qualifications and Credentials Framework (NZQCF), which comprises 10 levels (Levels 1-10) of increasing complexity and depth of knowledge. We added a Level 0 category to represent no formal qualification, resulting in an 11-category ordinal measure (0-10). The levels are defined as follows: Level 0 represents no formal qualification. Levels 1-3 cover basic to operational knowledge. Level 4 covers broader operational and theoretical knowledge. Level 5 covers broad operational or technical knowledge within a specific field. Level 6 covers specialised technical or theoretical knowledge with depth. Level 7 includes bachelor's degrees, graduate certificates, and graduate diplomas. Level 8 includes bachelor honours degrees, postgraduate certificates, and postgraduate diplomas. Level 9 represents master's degrees. Level 10 represents doctoral degrees. To ensure adequate cell counts and satisfy the positivity assumption required for causal inference, we coarsened the 11-category measure (0-10) into seven ordinal categories: no qualification (Level 0), certificates Levels 1-4 (foundation through broader vocational qualifications), certificates Levels 5-6 (advanced certificates and diplomas), bachelor's degree (Level 7), postgraduate qualifications (Level 8), master's degree (Level 9), and doctorate (Level 10). This coarsening preserves meaningful educational gradients while ensuring sufficient sample sizes across all covariate strata. In our statistical models, these ordinal categories are represented as binary indicators [1].

Items:

- What is your highest level of qualification?

Employed

Binary response: (0 = No, 1 = Yes) [5].

Items:

- Are you currently employed (This includes self-employed of casual work)?

Ethnicity

Coded string: (1 = New Zealand European; 2 = Māori; 3 = Pacific; 4 = Asian) [5].

Items:

- Which ethnic group(s) do you belong to?

Extraversion

Mini-IPIP6 Extraversion dimension: (i) I am the life of the party. (ii) I don't talk a lot. (r) (iii) I keep in the background. (r) (iv) I talk to a lot of different people at parties [2].

Items:

- I am the life of the party.
- I don't talk a lot (reversed).
- I keep in the background (reversed).
- I talk to a lot of different people at parties.

Honesty-Humility

Mini-IPIP6 Honesty-Humility dimension: (i) I feel entitled to more of everything. (r) (ii) I deserve more things in life. (r) (iii) I would like to be seen driving around in a very expensive car. (r) (iv) I would get a lot of pleasure from owning expensive luxury goods. (r) [2].

Items:

- I feel entitled to more of everything (reversed).
- I deserve more things in life (reversed).
- I would like to be seen driving around in a very expensive car (reversed).
- I would get a lot of pleasure from owning expensive luxury goods (reversed).

Hours of Charity (log)

Numerical: open-ended response [2].

Items:

- Hours spent … voluntary/charitable work.

Hours of Exercise (log)

Hours spent exercising in a typical week. Numerical: open-ended response [1].

Items:

- 'Hours spent…exercising/physical activity' (coded numeric 1-5)

Household Income (log)

- Please estimate your total household income (before tax) for the year XXXX.

Kessler Latent Anxiety

The anxiety subscale of the Kessler-6 (K6) psychological distress screening scale, comprising three items that capture physiological arousal, cognitive load, and restlessness (alpha = 0.72) [6].

Items:

- During the past 30 days, how often did…you feel nervous?
- During the past 30 days, how often did…you feel restless or fidgety?
- During the past 30 days, how often did…you feel that everything was an effort?

Kessler Latent Depression

The depression subscale of the Kessler-6 (K6) psychological distress screening scale, comprising three items that assess depressed mood, hopelessness, and worthlessness (alpha = 0.86) [6].

Items:

- During the past 30 days, how often did…you feel hopeless?
- During the past 30 days, how often did…you feel so depressed nothing could cheer you up?
- During the past 30 days, how often did…you feel worthless?

Male

Here, we coded all those who responded as Male as 1, and those who did not as 0 [7].

Items:

- We asked participants' gender in an open-ended question: "what is your gender?"

Neuroticism

Mini-IPIP6 Neuroticism dimension: (i) I have frequent mood swings. (ii) I am relaxed most of the time. (r) (iii) I get upset easily. (iv) I seldom feel blue. (r) [2].

Items:

- I have frequent mood swings.
- I am relaxed most of the time (reversed).
- I get upset easily.
- I seldom feel blue (reversed).

NZ Deprivation Index

Numerical: (1-10) [8].

Items:

- New Zealand Deprivation - Decile Index - Using 2018 Census Data

Socioeconomic Index (NZSEI)

This index uses the income, age, and education of a reference group, in this case, the 2013 New Zealand census, to calculate a score for each occupational group. Scores range from 10 (Lowest) to 90 (Highest). This list of index scores for occupational groups was used to assign each participant a NZSEI-13 score based on their occupation [9].

Items:

- We assessed occupational prestige and status using the New Zealand Socio-economic Index 13 (NZSEI-13).

Openness

Mini-IPIP6 Openness to Experience dimension: (i) I have a vivid imagination. (ii) I have difficulty understanding abstract ideas. (r) (iii) I do not have a good imagination. (r) (iv) I am not interested in abstract ideas. (r) [2].

Items:

- I have a vivid imagination.
- I have difficulty understanding abstract ideas (reversed).
- I do not have a good imagination (reversed).
- I am not interested in abstract ideas (reversed).

Parent

Parents were coded as 1, while the others were coded as 0 [1].

Items:

- If you are a parent, in which year was your eldest child born?

Partner

Coded as binary (has partner = 1) [1].

Items:

- What is your relationship status? (e.g., single, married, de-facto, civil union, widowed, living together, etc.)

Political Conservatism

- Please rate how politically liberal versus conservative you see yourself as being.

Religious Identification

"Do you identify with a religion and/or spiritual group?" If no, coded 1; if yes, "How important is your religion to how you see yourself?" measured on a 1 (Not Important) to 7 (Very Important) scale [1].

Items:

- How important is your religion to how you see yourself?

Rural (GCH 2018)

"Participants residence locations were coded according to a five-level ordinal categorisation ranging from Urban to Rural." [10].

Items:

- High Urban Accessibility = 1, Medium Urban Accessibility = 2, Low Urban Accessibility = 3, Remote = 4, Very Remote = 5.

Short Form Health

- In general, would you say your health is...

Social Support

- There are people I can depend on to help me if I really need it.
- There is no one I can turn to for guidance in times of stress (reversed).
- I know there are people I can turn to when I need help.

Hours with Children (log)

Participants reported the number of hours they spent with children in the previous week. We used the log-transformed version for modelling.

Hours Commuting (log)

Participants reported the number of hours they spent commuting in the previous week. We used the log-transformed version for modelling.

Hours of Housework (log)

Participants reported the number of hours they spent on housework or cooking in the previous week. We used the log-transformed version for modelling.

Exposure

Hours of Work Per Week

Weekly hours of paid work were measured by an open-ended item asking participants to estimate time spent in paid employment in the previous week.

Outcomes

Alcohol Frequency

Participants could chose between the following responses: '(1 = Never - I don't drink, 2 = Monthly or less, 3 = Up to 4 times a month, 4 = Up to 3 times a week, 5 = 4 or more times a week, 6 = Don't know)' [11].

Items:

- "How often do you have a drink containing alcohol?"

Alcohol Intensity

Participants responded using an open-ended box [11].

Items:

- "How many drinks containing alcohol do you have on a typical day when drinking alcohol? (number of drinks on a typical day when drinking)"

Social Belonging

We assessed felt belongingness with three items adapted from the Sense of Belonging Instrument (Hagerty & Patusky, 1995): (1) "Know that people in my life accept and value me"; (2) "Feel like an outsider"; (3) "Know that people around me share my attitudes and beliefs". Participants responded on a scale from 1 (Very Inaccurate) to 7 (Very Accurate). The second item was reversely coded [3].

Items:

- Know that people in my life accept and value me.
- Feel like an outsider (reversed).
- Know that people around me share my attitudes and beliefs.

Body Satisfaction

Ordinal response [12].

Items:

- I am satisfied with the appearance, size and shape of my body.

Gratitude

Ordinal response scale 1 = Strongly Disagree to 7 = Strongly Agree [13].

Items:

- I have much in my life to be thankful for.
- When I look at the world, I don't see much to be grateful for (reversed).
- I am grateful to a wide variety of people.

BMI

Based on participants indication of their height and weight we calculated the BMI by dividing the weight in kilograms by the square of the height in meters [1].

Items:

- What is your height? (metres)" and "What is your weight? (kg).

Fatigue

A single item assessing subjective fatigue over the past 30 days, scored on the same five-point frequency scale as the Kessler-6 items (0 = None of the Time to 4 = All of the Time). Introduced in the NZAVS at Time 5 [14].

Items:

- During the last 30 days, how often did … you feel exhausted?

Hours of Sleep

Open ended response [15].

Items:

- During the past month, on average, how many hours of actual sleep did you get per night?

Hours of Exercise (log)

Hours spent exercising in a typical week. Numerical: open-ended response [1].

Items:

- 'Hours spent…exercising/physical activity' (coded numeric 1-5)

Kessler Latent Anxiety

The anxiety subscale of the Kessler-6 (K6) psychological distress screening scale, comprising three items that capture physiological arousal, cognitive load, and restlessness (alpha = 0.72) [6].

Items:

- During the past 30 days, how often did…you feel nervous?
- During the past 30 days, how often did…you feel restless or fidgety?
- During the past 30 days, how often did…you feel that everything was an effort?

Kessler Latent Depression

The depression subscale of the Kessler-6 (K6) psychological distress screening scale, comprising three items that assess depressed mood, hopelessness, and worthlessness (alpha = 0.86) [6].

Items:

- During the past 30 days, how often did…you feel hopeless?
- During the past 30 days, how often did…you feel so depressed nothing could cheer you up?
- During the past 30 days, how often did…you feel worthless?

Life Satisfaction

[16].

Items:

- I am satisfied with my life.
- In most ways my life is close to ideal.

Meaning: Purpose

A single item from the Meaning in Life Questionnaire (MLQ) assessing presence of purpose, scored on a seven-point [17].

Response scale: 1 = Strongly Disagree to 7 = Strongly Agree

Items:

- My life has a clear sense of purpose

Meaning: Sense

A single item from the Meaning in Life Questionnaire (MLQ) assessing presence of meaning, scored on a seven-point [17].

Response scale: 1 = Strongly Disagree to 7 = Strongly Agree

Items:

- I have a good sense of what makes my life meaningful.

Neighbourhood Community

[18].

Items:

- I feel a sense of community with others in my local neighbourhood.

Perfectionism

[19].

Items:

- Doing my best never seems to be enough.
- My performance rarely measures up to my standards.
- I am hardly ever satisfied with my performance.

PWB: Standard of Living

[20].

Items:

- Please rate your level of satisfaction with the following aspects of your life...Your standard of living.

PWB: Future Security

[20].

Items:

- Please rate your level of satisfaction with the following aspects of your life...Your future security.

PWB: Health

[20].

Items:

- Please rate your level of satisfaction with the following aspects of your life...Your health.

PWB: Relationships

[20].

Items:

- Please rate your level of satisfaction with the following aspects of your life...Your personal relationships.

Rumination

Ordinal responses: 0 = None of The Time, 1 = A little of The Time, 2 = Some of The Time, 3 = Most of The Time, 4 = All of The Time [21].

Items:

- During the last 30 days, how often did...you have negative thoughts that repeated over and over?

Self-Control: Have Lots

[22].

Items:

- In general, I have a lot of self-control.

Self-Control: Wish More (Reversed)

[22].

Items:

- I wish I had more self-discipline.

Self-Esteem

[23].

Items:

- On the whole am satisfied with myself.
- Take a positive attitude toward myself.
- Am inclined to feel that I am a failure (reversed).

Sexual Satisfaction

Participants were asked to report their sexual orientation; ordinal response: 1 = Not satisfied to 7 = Very satisfied [1].

Items:

- How satisfied are you with your sex life?

Short Form Health

- In general, would you say your health is…

Social Support

- There are people I can depend on to help me if I really need it.
- There is no one I can turn to for guidance in times of stress (reversed).
- I know there are people I can turn to when I need help.

Vengefulness

This scale contains three items, adapted from [24] and [25], and developed for NZAVS; [1].

Items:

- Sometimes I can't sleep because of thinking about past wrongs I have suffered.
- I can usually forgive and forget when someone does me wrong (reversed scored).
- I find myself regularly thinking about past times that I have been wronged.

Supplement S3: Identification Assumptions, Missing Data, and Cohort Support

Three identification assumptions support a causal interpretation of the modified treatment policy contrasts [26,27]. **Consistency** means that the observed outcome under the observed exposure equals the counterfactual outcome under that same exposure value. For a continuous exposure such as work hours, this requires the shift in hours to represent a sufficiently coherent intervention even though hour changes can arise through different social processes [28]. A related no-interference condition requires one participant's work hours not to affect another participant's well-being through the same causal mechanism. This seems plausible for these self-reported outcomes, although workplace interdependencies (for example, one colleague's overtime redistributing tasks to others) could in principle violate it.

Conditional exchangeability means that, after conditioning on the measured baseline history, the observed exposure assignment at T_1 is as good as random. The richly adjusted baseline block strengthens this assumption relative to cross-sectional designs, but unmeasured confounders that operate after baseline remain possible. **Positivity** means that people with similar measured histories still exhibit adequate variation in work hours. We refer to this empirical side of positivity as "overlap", because the shifted policies must remain supported by observed data rather than learned only by extrapolation.

Confounding control followed the modified disjunctive cause criterion [29]. The baseline adjustment set includes 34 variables covering demographics, socioeconomic position, personality, health, health behaviours, and time use, along with baseline work hours and baseline values of all 28 outcomes. Because the intervention occurs only at

T_1, we conditioned only on baseline history. We did not adjust for variables measured at or after the exposure wave, because post-exposure adjustment can introduce bias rather than remove it [30,31]. The main-text causal DAG represents this structure.

We handled missing data in two stages. We imputed baseline covariates and baseline outcomes with missing values after reshaping to wide format. We did not impute exposure-wave work hours or outcome-wave outcomes. Instead, we treated non-observation after baseline as censoring and handled it through inverse probability of censoring weighting, targeting the mean outcome that would have been observed in the baseline cohort under each policy had censoring not occurred. Estimation used `lmtp::lmtp_tmle` via `margot::margot_lmtp()`, with a Super Learner library comprising `SL.mean`, `SL.ranger`, `SL.xgboost`, and `SL.glmnet`, five-fold cross-fitting, and 99th-percentile trimming of the baseline survey weights.

Table S4 records the eligibility audit for the baseline-defined cohort.

Table 4. Table S4. Eligibility audit for the baseline-defined cohort.

criterion	n
Observed at baseline wave (T0)	38550
Aged 18 to 60 at baseline	25313
Observed baseline work hours	37437
Observed baseline employment status	38334
Observed baseline survey weight	38550
Eligible baseline cohort	24579

The analysis begins with 38,550 respondents observed at the baseline wave. Applying the pre-retirement age restriction (18 to 60 years), requiring observed baseline work hours, observed baseline employment status, and observed baseline survey weights yields an eligible baseline cohort of 24,579 participants.

Table S5 reports the observed rows available at each wave within this fixed baseline cohort.

Table 5. Table S5. Observed rows by wave within the fixed baseline cohort.

wave	rows_in_baseline_cohort	observed_rows	observed_pct
T0 (baseline, 2020/2021)	24579	24579	100.0%
T1 (exposure, 2021/2022)	24579	17634	71.7%
T2 (outcome, 2022/2023)	24579	15275	62.1%

Within the baseline-defined cohort, 71.7% of participants are observed at the exposure wave and 62.1% are observed at the outcome wave. These are not revised eligibility rules. They describe the censoring pattern that the target-trial estimands must address.

Table S5a reports outcome-wave availability for the 28 well-being outcomes in the baseline-defined cohort.

Table 6. Table S5a. Outcome-wave availability for the 28 well-being outcomes.

Outcome	Observed n	Missing %
meaning_purpose	14410	5.7%
sexual_satisfaction	14526	4.9%
self_control_have_lots	14583	4.5%
bodysat	14645	4.1%
hlth_sleep_hours	14654	4.1%
meaning_sense	14648	4.1%
neighbourhood_community	14645	4.1%
self_control_wish_more_reversed	14682	3.9%
alcohol_intensity	14771	3.3%
hours_exercise_log	14809	3.1%
lifesat	14860	2.7%
perfectionism	15169	0.7%
alcohol_frequency	15180	0.6%
vengeful_rumin	15187	0.6%
belong	15201	0.5%
hlth_bmi	15204	0.5%
hlth_fatigue	15203	0.5%
pwb_your_health	15195	0.5%
rumination	15200	0.5%
pwb_your_future_security	15209	0.4%
pwb_your_relationships	15207	0.4%
self_esteem	15214	0.4%
support	15211	0.4%
gratitude	15230	0.3%
pwb_standard_living	15229	0.3%
short_form_health	15235	0.3%
kessler_latent_anxiety	15237	0.2%
kessler_latent_depression	15237	0.2%

Supplement S4: Formal Policy Definitions and Censoring Intervention

Let A_1 denote observed weekly work hours at the exposure wave, let W_0 denote the measured baseline history used for adjustment, and let Y_2 denote a generic well-being outcome measured one wave later. In this study, W_0 includes baseline covariates, baseline work hours, and the baseline value of every outcome. A deterministic modified treatment policy is a rule $d(\cdot)$ that maps the observed exposure value at T_1 to a policy-assigned value.

For observed work hours $a \geq 0$, the three policies are:

$$d^{+10}(a) = a + 10,$$

$$d^{-10}(a) = \max(a - 10, 0),$$

and

$$d^{0}(a) = a.$$

The corresponding causal contrasts compare each shifted policy with the identity policy:

$$\mathbb{E}(Y_2^{d^{+10}}) - \mathbb{E}(Y_2^{d^0}) \qquad \text{and} \qquad \mathbb{E}(Y_2^{d^{-10}}) - \mathbb{E}(Y_2^{d^0}).$$

Attrition is handled through an additional counterfactual intervention. Let R_2^d indicate whether a participant would remain uncensored through outcome measurement under policy d. The no-censoring intervention sets $R_2^d \equiv 1$. The observed course corrected for attrition is therefore the identity work-hours policy combined with the no-censoring intervention, so that the reported contrasts target the baseline cohort rather than the selected subset observed at the end of follow-up.

The estimator relies on three nuisance functions. Let g denote the conditional density of the exposure at T_1 given baseline history, let c denote the conditional probability of remaining uncensored at each wave given prior history, and let m denote the conditional outcome regression at T_2. The modified treatment policy estimator, implemented with `lmtp`, combines these to recover policy-specific marginal means under the doubly robust property: the estimate is consistent if either the outcome model m or both the exposure model g and censoring model c are correctly specified [32,33]. We estimated g, c, and m with five-fold cross-fitted Super Learner ensembles comprising a simple mean (`SL.mean`), random forests (`SL.ranger`), gradient boosting (`SL.xgboost`), and regularised regression (`SL.glmnet`) [34]. This strategy reduces dependence on any single parametric specification, but the approach still depends on the causal design assumptions and adequate positivity.

In implementation, the treatment history passed to `lmtp` was the two-node vector (A_0, A_1), but we applied the intervention only at T_1. The identity policy was internally computed in `lmtp`, so the reported causal estimands are contrasts between each policy-specific mean and the observed-course mean under complete follow-up.

Supplement S5: Positivity Diagnostics

We assessed positivity for the two deterministic shift policies (+10 hours, -10 hours) and the identity observed-course policy. The `lmtp` estimator reweights observed person-time using density ratios that compare the policy-modified and observed exposure processes. Weak "overlap" appears when many uncensored rows receive very small products of ratios, meaning the estimate depends on a narrow subset of trajectories. Very large ratios also signal limited support because they inflate variance. For that reason, we also report effective sample sizes after right-tail trimming at the 99th percentile.

Table 7. Table S6. Positivity diagnostics summary by policy.

Shift	Support	Zero %	Outside [0.1000, 10.0000]	Prod < 0.1000	Prod > 10.0000	Cum ESS
Observed (Censoring-Adjusted)	Adequate	59.132	0.000	0.000	0.000	9922.227
Loss 10	Adequate	59.132	3.829	3.829	0.000	4123.102
Gain 10	Caution	59.132	23.227	19.971	3.256	2110.458

Under the +10-hour policy, 76.8% of uncensored rows remained within the

product-of-ratio interval [0.1, 10], while 20.0% fell below 0.1 and 3.3% exceeded 10, indicating materially weaker support for the upward-shift intervention. Under the -10-hour policy, 96.2% remained within [0.1, 10], and only 3.8% fell below 0.1. After trimming, cumulative effective sample sizes were approximately 2,110 for the +10-hour policy, 4,123 for the -10-hour policy, and 9,922 for the identity policy in the baseline-defined cohort (N = 24,579).

Supplement S6: Numerical Intervention Contrasts on the Standardised and Original Scales

Tables S6a and S6b report the Bonferroni-corrected modified treatment policy contrasts on the standardised outcome scale. Tables S6c and S6d back-transform those same contrasts and interval bounds to the original outcome scale by multiplying each estimate by the observed outcome-wave standard deviation for that outcome. Sensitivity statistics (E-values) are only reported for the standardised tables because the sensitivity analysis was conducted on that common effect scale.

Table 8. Table S6a. Standardised effects: +10 hours vs observed course (Bonferroni-corrected).

Outcome	ATE	Lower CI	Upper CI	E-Value	E-Value Bound
Fatigue	0.079	0.035	0.123	1.358	1.216
PWB Your Health	-0.055	-0.100	-0.010	1.284	1.103
Hours of Sleep	-0.052	-0.101	-0.003	1.274	1.052
Support	0.047	0.002	0.092	1.257	1.041
Rumination	0.047	-0.010	0.104	1.257	1.000
Short Form Health	-0.040	-0.085	0.005	1.233	1.000
Perfectionism	0.033	-0.020	0.086	1.208	1.000
PWB Your Future Security	-0.032	-0.079	0.015	1.204	1.000
BMI	0.030	0.006	0.054	1.196	1.081
Body Satisfaction	-0.030	-0.077	0.017	1.196	1.000
Log Hours of Exercise	-0.029	-0.080	0.022	1.192	1.000
Gratitude	0.024	-0.025	0.073	1.172	1.000
Neighbourhood Community	-0.023	-0.071	0.025	1.168	1.000
Meaning Sense	-0.022	-0.086	0.042	1.164	1.000
Kessler Latent Depression	0.021	-0.030	0.072	1.160	1.000
Meaning Purpose	-0.020	-0.075	0.035	1.155	1.000
Self-Control: Have Lots	-0.019	-0.062	0.024	1.151	1.000
Alcohol Frequency	0.013	-0.023	0.049	1.122	1.000
Vengefulness	0.012	-0.040	0.064	1.116	1.000
Alcohol Intensity	-0.012	-0.071	0.047	1.116	1.000
PWB Your Relationships	0.007	-0.038	0.052	1.087	1.000
Kessler Latent Anxiety	-0.006	-0.051	0.039	1.080	1.000
Sexual Satisfaction	0.004	-0.043	0.051	1.064	1.000
Self-Esteem	-0.003	-0.050	0.044	1.055	1.000
Self-Control: Wish More (Reversed)	0.003	-0.042	0.048	1.055	1.000
PWB Standard of Living	0.002	-0.051	0.055	1.045	1.000
Life Satisfaction	-0.002	-0.055	0.051	1.045	1.000
Belonging	0.001	-0.044	0.046	1.031	1.000

Table 9. Table S6b. Standardised effects: -10 hours vs observed course (Bonferroni-corrected).

Outcome	ATE	Lower CI	Upper CI	E-Value	E-Value Bound
Fatigue	-0.042	-0.069	-0.015	1.240	1.132
PWB Your Health	0.030	0.003	0.057	1.196	1.055
Sexual Satisfaction	-0.022	-0.051	0.007	1.164	1.000
PWB Standard of Living	0.022	-0.007	0.051	1.164	1.000
Neighbourhood Community	0.022	-0.004	0.048	1.164	1.000
Log Hours of Exercise	0.022	-0.009	0.053	1.164	1.000
Hours of Sleep	0.022	-0.006	0.050	1.164	1.000
BMI	-0.020	-0.034	-0.006	1.155	1.083
Rumination	-0.018	-0.050	0.014	1.146	1.000
Body Satisfaction	0.018	-0.008	0.044	1.146	1.000
Support	0.017	-0.007	0.041	1.141	1.000
Life Satisfaction	-0.017	-0.044	0.010	1.141	1.000
Short Form Health	0.016	-0.008	0.040	1.137	1.000
Belonging	-0.015	-0.041	0.011	1.132	1.000
Meaning Sense	-0.014	-0.047	0.019	1.127	1.000
Gratitude	0.013	-0.012	0.038	1.122	1.000
Self-Control: Have Lots	0.011	-0.016	0.038	1.111	1.000
PWB Your Future Security	0.011	-0.017	0.039	1.111	1.000
Perfectionism	-0.010	-0.035	0.015	1.105	1.000
Self-Esteem	0.009	-0.015	0.033	1.099	1.000
Self-Control: Wish More (Reversed)	-0.009	-0.032	0.014	1.099	1.000
Vengefulness	0.007	-0.018	0.032	1.087	1.000
Kessler Latent Anxiety	-0.006	-0.034	0.022	1.080	1.000
Alcohol Intensity	-0.006	-0.041	0.029	1.080	1.000
Kessler Latent Depression	-0.005	-0.036	0.026	1.072	1.000
Meaning Purpose	-0.004	-0.035	0.027	1.064	1.000
Alcohol Frequency	-0.004	-0.024	0.016	1.064	1.000
PWB Your Relationships	-0.001	-0.030	0.028	1.031	1.000

Table 10. Table S6c. Original-scale effects: +10 hours vs observed course.

Outcome	ATE	Lower CI	Upper CI
Fatigue	0.085	0.038	0.133
PWB Your Health	-0.132	-0.240	-0.024
Hours of Sleep	-0.057	-0.110	-0.003
Support	0.056	0.002	0.109
Rumination	0.047	-0.010	0.104
Short Form Health	-0.047	-0.100	0.006
Perfectionism	0.048	-0.029	0.126
PWB Your Future Security	-0.080	-0.198	0.038
BMI	0.188	0.038	0.338
Body Satisfaction	-0.051	-0.131	0.029
Log Hours of Exercise	-0.024	-0.065	0.018
Gratitude	0.022	-0.023	0.066
Neighbourhood Community	-0.037	-0.113	0.040
Meaning Sense	-0.028	-0.108	0.053
Kessler Latent Depression	0.016	-0.023	0.055

Table 10. Table S6c. Original-scale effects: +10 hours vs observed course.

Outcome	ATE	Lower CI	Upper CI
Meaning Purpose	-0.029	-0.110	0.051
Self-Control: Have Lots	-0.028	-0.091	0.035
Alcohol Frequency	0.017	-0.030	0.065
Vengefulness	0.015	-0.052	0.083
Alcohol Intensity	-0.023	-0.134	0.089
PWB Your Relationships	0.016	-0.088	0.120
Kessler Latent Anxiety	-0.005	-0.040	0.030
Sexual Satisfaction	0.007	-0.077	0.091
Self-Esteem	-0.004	-0.068	0.059
Self-Control: Wish More (Reversed)	0.005	-0.075	0.086
PWB Standard of Living	0.004	-0.109	0.117
Life Satisfaction	-0.003	-0.070	0.065
Belonging	0.001	-0.050	0.052

Table 11. Table S6d. Original-scale effects: -10 hours vs observed course.

Outcome	ATE	Lower CI	Upper CI
Fatigue	-0.045	-0.075	-0.016
PWB Your Health	0.072	0.007	0.137
Sexual Satisfaction	-0.039	-0.091	0.012
PWB Standard of Living	0.047	-0.015	0.109
Neighbourhood Community	0.035	-0.006	0.076
Log Hours of Exercise	0.018	-0.007	0.043
Hours of Sleep	0.024	-0.007	0.054
BMI	-0.125	-0.213	-0.038
Rumination	-0.018	-0.050	0.014
Body Satisfaction	0.031	-0.014	0.075
Support	0.020	-0.008	0.049
Life Satisfaction	-0.022	-0.056	0.013
Short Form Health	0.019	-0.009	0.047
Belonging	-0.017	-0.047	0.013
Meaning Sense	-0.018	-0.059	0.024
Gratitude	0.012	-0.011	0.035
Self-Control: Have Lots	0.016	-0.023	0.056
PWB Your Future Security	0.028	-0.043	0.098
Perfectionism	-0.015	-0.051	0.022
Self-Esteem	0.012	-0.020	0.045
Self-Control: Wish More (Reversed)	-0.016	-0.057	0.025
Vengefulness	0.009	-0.023	0.041
Kessler Latent Anxiety	-0.005	-0.026	0.017
Alcohol Intensity	-0.011	-0.078	0.055
Kessler Latent Depression	-0.004	-0.028	0.020
Meaning Purpose	-0.006	-0.051	0.039
Alcohol Frequency	-0.005	-0.032	0.021
PWB Your Relationships	-0.002	-0.069	0.065

Supplement S7: Descriptive Baseline Cross-Sectional Associations

For contrast with the target-trial estimates, we also fit a naive cross-sectional regression relating baseline work hours to baseline well-being outcomes within the eligible cohort. This comparison is purely descriptive. It avoids post-baseline selection because both variables are measured at the eligibility wave, but it does not emulate an intervention, does not address reverse causation, and we do not interpret it through a causal sensitivity analysis.

Table 12. Table S7. Descriptive baseline cross-sectional associations between baseline work hours and baseline w[illegible] outcomes (standardised scale).

Outcome	ATE	2.5 %	97.5 %
Alcohol Frequency	0.059	0.052	0.065
Kessler Latent Depression	-0.054	-0.060	-0.047
PWB Your Health	0.048	0.042	0.055
Short Form Health	0.045	0.038	0.051
Self-Esteem	0.044	0.038	0.051
Rumination	-0.040	-0.046	-0.033
Meaning Purpose	0.037	0.030	0.043
Vengefulness	-0.036	-0.043	-0.030
Alcohol Intensity	0.035	0.029	0.042
Log Hours of Exercise	-0.032	-0.039	-0.026
PWB Your Future Security	0.032	0.025	0.038
Kessler Latent Anxiety	-0.031	-0.037	-0.024
Life Satisfaction	0.028	0.022	0.035
PWB Standard of Living	0.028	0.021	0.035
Hours of Sleep	-0.027	-0.034	-0.021
Self-Control: Have Lots	0.027	0.021	0.034
Perfectionism	-0.025	-0.031	-0.018
Belonging	0.024	0.018	0.031
Neighbourhood Community	-0.019	-0.025	-0.012
Self-Control: Wish More (Reversed)	0.017	0.011	0.024
PWB Your Relationships	0.016	0.010	0.023
BMI	0.010	0.004	0.017
Fatigue	-0.004	-0.010	0.003
Support	0.004	-0.003	0.010
Meaning Sense	-0.003	-0.010	0.003
Body Satisfaction	0.002	-0.004	0.009
Gratitude	0.000	-0.006	0.007
Sexual Satisfaction	0.000	-0.007	0.006

The descriptive pattern is broader than the supported modified treatment policy contrasts in the main text. It also points in substantively different directions, making many baseline coefficients look meaningful while missing fatigue and support and showing only a slight positive slope for BMI. This attenuation is one of the clearest reasons to prefer explicitly stated causal contrasts over contemporaneous association models.

Supplement S8: TARGET Checklist

We report this study following the TARGET (Transparent Reporting of Observational Studies Emulating a Target Trial) statement [35]. The checklist below maps each TARGET item to the corresponding section of the manuscript and supplement.

No.	Item	Reported in
1	Identify that the study emulates a target trial using observational data; state objectives and briefly summarise the target trial	Abstract; Introduction
2	Report the data source(s) used for emulation	Abstract; Method: Sample
3	Summarise key assumptions, statistical methods, findings, and conclusions	Abstract
	**Introduction**	
4	Describe the scientific background and gap in knowledge	Introduction
5	Summarise the causal question	Introduction; Method: Target trial
6	Describe the rationale for emulating a target trial	Introduction
	**Methods**	
7	Cite data sources; describe original purpose, type, geographic location, setting, and time period	Method: Sample
8a	Eligibility criteria and how they were operationalised	Method: Sample; Supplement S3
8b	Treatment strategies and how they were operationalised	Method: Target trial; Supplement S4
8c	Assignment procedures and how assignment was operationalised	Method: Target trial; Method: Confounding control; Method: Estimation

No.	Item	Reported in
8d	Follow-up: clarify that follow-up starts at assignment; describe how operationalised	Method: Target trial; Method: Exposure and outcomes; Supplement S3
8e	Outcomes and how they were operationalised	Method: Exposure and outcomes; Supplement S2
8f	Causal contrasts and how they were operationalised	Method: Target trial; Supplement S4
8g	Identifying assumptions for each causal estimand; describe variables related to these assumptions	Method: Confounding control; Method: Estimation; Supplement S3; Supplement S5
8h	Data analysis procedures for each causal estimand	Method: Estimation; Supplement S6
8i	Additional analyses for each causal estimand	Results: Positivity and empirical support; Supplement S5–S7
	**Results**	
9	Participant selection: report numbers assessed, eligible, and assigned	Method: Sample; Supplement S3
10	Baseline characteristics by treatment strategy	Supplement S1; strategies are counterfactual policies applied to the same baseline cohort
11	Follow-up: summarise length and describe reasons for end of follow-up	Method: Sample; Method: Exposure and outcomes; Supplement S3
12	Missing data: describe frequency by variable	Method: Estimation; Supplement S3; Table S5a
13	Outcomes: describe frequency or distribution at each wave	Method: Exposure and outcomes; Supplement S1
14	Effect estimates for each causal contrast, with measures of precision	Results; Supplement S6

No.	Item	Reported in
15	Additional analyses: report sensitivity of estimates to choices in operationalisation, assumptions, and analysis	Results: Positivity and empirical support; Supplement S5–S7
	**Discussion**	
16	Provide an interpretation of the key findings	Discussion
17	Discuss limitations, including differences between target trial and emulation, and plausibility of assumptions	Discussion
	**Other information**	
18	Ethics approval	Method: Sample
19	Registration	Method: Sample
20	Data and code availability	Data availability statement
21	Funding sources	Not separately reported; CRediT authorship contribution statement notes funding acquisition roles
22	Conflicts of interest	Declaration of competing interest (none declared)

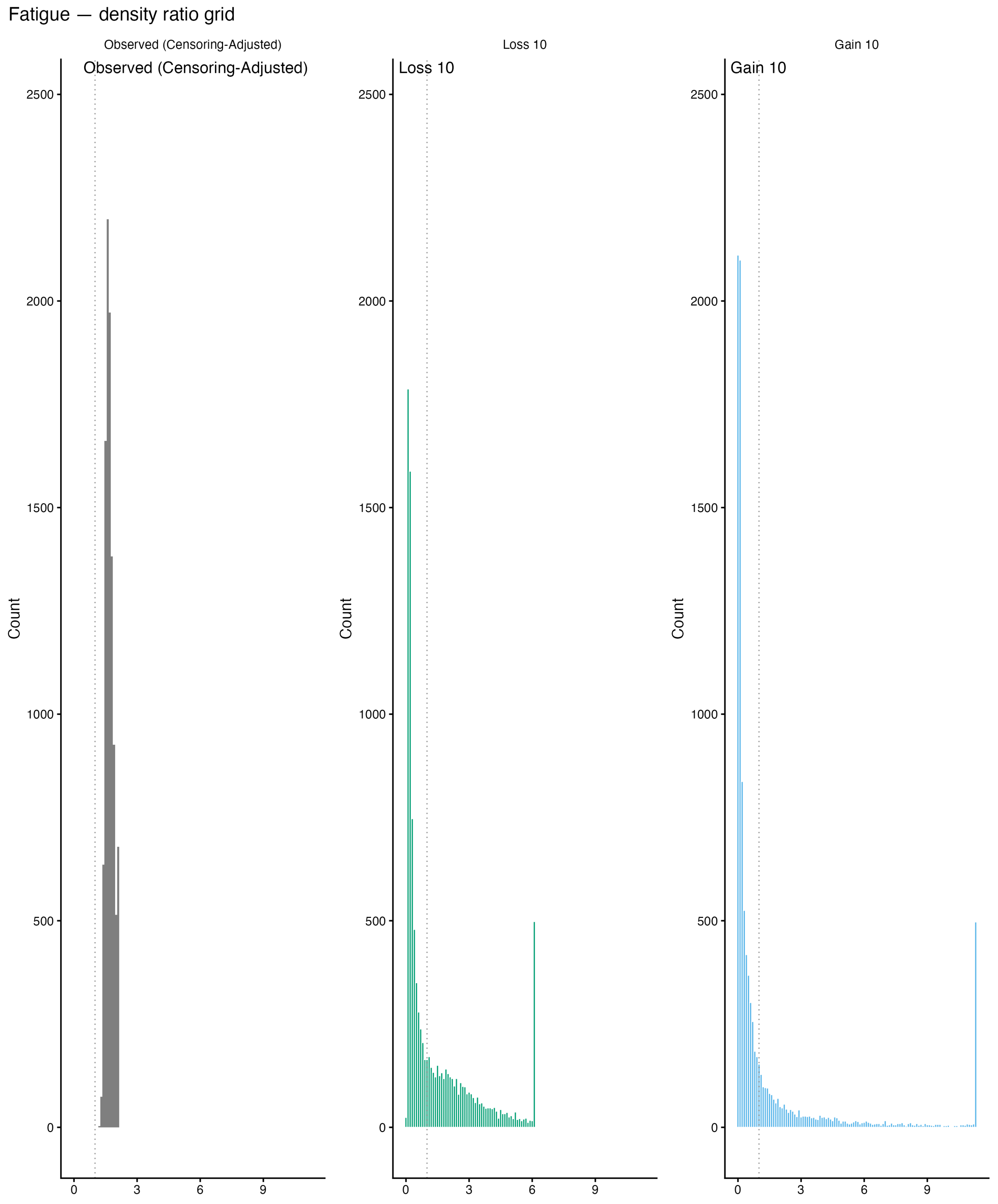

Figure 1. Figure S1. Density-ratio overlap diagnostics for the primary policies.

References

1. Sibley CG (2021) Sampling procedure and sample details for the New Zealand Attitudes and Values Study. doi:10.31234/osf.io/wgqvy

2. Sibley CG, Luyten N, Purnomo M, Mobberley A, Wootton LW, et al. (2011) The mini-IPIP6: Validation and extension of a short measure of the big-six factors of personality in New Zealand. New Zealand Journal of Psychology 40: 142–159.

3. Hagerty BMK, Patusky K (1995) Developing a measure of sense of belonging: Nursing Research 44: 9–13. Available: http://journals.lww.com/00006199-199501000-00003.

4. Hoverd WJ, Sibley CG (2010) Religious and denominational diversity in New Zealand 2009. New Zealand Sociology 25: 59–87.

5. Statistics New Zealand (2017) Statistical standard for geographic areas 2018 (SSGA18). Wellington, New Zealand: Statistics New Zealand. Available: https://www.stats.govt.nz/methods/statistical-standard-for-geographic-areas-2018/.

6. Kessler RC, Andrews G, Colpe LJ, Hiripi E, Mroczek DK, et al. (2002) Short screening scales to monitor population prevalences and trends in non-specific psychological distress. Psychological Medicine 32: 959–976. Available: https://www.cambridge.org/core/product/identifier/S0033291702006074/type/journal_article.

7. Fraser G, Bulbulia J, Greaves LM, Wilson MS, Sibley CG (2020) Coding responses to an open-ended gender measure in a New Zealand national sample. The Journal of Sex Research 57: 979–986. Available: https://doi.org/10.1080/00224499.2019.1687640. Accessed 27 Nov 2022.

8. Atkinson J, Salmond C, Crampton P (2019) NZDep2018 index of deprivation, user's manual. Wellington.

9. Fahy KM, Lee A, Milne BJ (2017) New Zealand socio-economic index 2013. Wellington, New Zealand: Statistics New Zealand-Tatauranga Aotearoa.

10. Whitehead J, Davie G, Graaf B de, Crengle S, Lawrenson R, et al. (2023) Unmasking hidden disparities: A comparative observational study examining the impact of different rurality classifications for health research in aotearoa new zealand. BMJ open 13: e067927.

11. Health Ministry of (2013) The New Zealand Health Survey: Content guide 2012-2013. Princeton University Press.

12. Stronge S, Greaves LM, Milojev P, West-Newman T, Barlow FK, et al. (2015) Facebook is linked to body dissatisfaction: Comparing users and non-users. Sex Roles 73: 200–213.

13. McCullough ME, Emmons RA, Tsang J-A (2002) The grateful disposition: A conceptual and empirical topography. Journal of Personality and Social Psychology 82: 112–127. doi:10.1037/0022-3514.82.1.112

14. Sibley CG, Afzali MU, Satherley N, Ejova A, Stronge S, et al. (2020) Prejudice toward muslims in New Zealand: Insights from the New Zealand Attitudes and Values Study. New Zealand Journal of Psychology 49.

15. Buysse DJ, Reynolds III CF, Monk TH, Berman SR, Kupfer DJ (1989) The pittsburgh sleep quality index: A new instrument for psychiatric practice and research. Psychiatry research 28: 193–213.

16. Diener E, Emmons RA, Larsen RJ, Griffin S (1985) The satisfaction with life scale. Journal of Personality Assessment 49: 71–75.

17. Steger MF, Frazier P, Oishi S, Kaler M (2006) The meaning in life questionnaire: Assessing the presence of and search for meaning in life. Journal of Counseling Psychology 53: 80–93. doi:10.1037/0022-0167.53.1.80

18. Sengupta NK, Luyten N, Greaves LM, Osborne D, Robertson A, et al. (2013) Sense of community in New Zealand neighbourhoods: A multi-level model predicting social capital. New Zealand Journal of Psychology 42: 36–45.

19. Rice KG, Richardson CME, Tueller S (2014) The short form of the revised almost perfect scale. Journal of Personality Assessment 96: 368–379. Available: https://doi.org/10.1080/00223891.2013.838172. Accessed 27 Nov 2022.

20. Cummins RA, Eckersley R, Pallant J, Vugt J van, Misajon R (2003) Developing a national index of subjective wellbeing: The australian unity wellbeing index. Social Indicators Research 64: 159–190. Available: https://doi.org/10.1023/A:1024704320683. Accessed 27 Nov 2022.

21. Nolen-hoeksema S, Morrow J (1993) Effects of rumination and distraction on naturally occurring depressed mood. Cognition and Emotion 7: 561–570. Available: https://doi.org/10.1080/02699939308409206. Accessed 27 Nov 2022.

22. Tangney JP, Baumeister RF, Boone AL (2004) High self-control predicts good adjustment, less pathology, better grades, and interpersonal success. J Pers 72: 271–324. doi:10.1111/j.0022-3506.2004.00263.x

23. Rosenberg M (1965) Rosenberg self-esteem scale (RSE). Acceptance and commitment therapy. Measures package 61: 18.

24. Caprara GV (1986) Indicators of aggression: The dissipation-rumination scale. Personality and Individual Differences 7: 763–769. Available: https://www.sciencedirect.com/science/article/pii/0191886986900747. Accessed 27 Nov 2022.

25. Berry JW, Worthington Jr. EL, O'Connor LE, Parrott III L, Wade NG (2005) Forgivingness, vengeful rumination, and affective traits. Journal of Personality 73: 183–226. Available: http://onlinelibrary.wiley.com/doi/abs/10.1111/j.1467-6494.2004.00308.x. Accessed 27 Nov 2022.

26. Hernan MA, Robins JM (2020) Causal inference: What if? Taylor & Francis. Available: https://www.hsph.harvard.edu/miguel-hernan/causal-inference-book/.

27. Bulbulia JA (2024) Methods in causal inference. Part 1: Causal diagrams and confounding. Evolutionary Human Sciences 6: e40.

28. VanderWeele TJ (2009) Concerning the consistency assumption in causal inference. Epidemiology 20: 880. Available: https://journals.lww.com/epidem/Fulltext/2009/11000/Concerning_the_Consistency_Assumption_in_Causal.18.aspx.

29. VanderWeele TJ (2019) Principles of confounder selection. European journal of epidemiology 34: 211–219.

30. VanderWeele TJ, Mathur MB, Chen Y (2020) Outcome-wide longitudinal designs for causal inference: A new template for empirical studies. Statistical Science 35: 437–466.

31. Montgomery JM, Nyhan B, Torres M (2018) How conditioning on posttreatment variables can ruin your experiment and what to do about it. American Journal of Political Science 62: 760–775. Available: https://onlinelibrary.wiley.com/doi/abs/10.1111/ajps.12357.

32. Díaz I, Williams N, Hoffman KL, Schenck EJ (2021) Non-parametric causal effects based on longitudinal modified treatment policies. Journal of the American Statistical Association. Available: https://doi.org/10.1080/01621459.2021.1955691.

33. Williams N, Díaz I (2023) Lmtp: An R package for estimating the causal effects of modified treatment policies. Observational Studies 9: 103–122.

34. Van Der Laan MJ, Rose S (2011) Targeted learning: Causal inference for observational and experimental data. New York, NY: Springer. Available: https://link.springer.com/10.1007/978-1-4419-9782-1.

35. Cashin AG, Hansford HJ, Hernán MA, et al. (2025) Transparent reporting of observational studies emulating a target trial—the TARGET statement. JAMA 334: 1084–1093. doi:10.1001/jama.2025.13350
